# Real-Life Experience with Oral Eliglustat in Patients with Gaucher Disease Previously Treated with Enzyme Replacement Therapy

**DOI:** 10.3390/jcm11216265

**Published:** 2022-10-24

**Authors:** Majdolen Istaiti, Michal Becker-Cohen, Tama Dinur, Shoshana Revel-Vilk, Ari Zimran

**Affiliations:** 1 Gaucher Unit, Shaare Zedek Medical Center, Jerusalem 9103102, Israel; 2 Faculty of Medicine, Hebrew University, Jerusalem 91120, Israel

**Keywords:** Gaucher disease, eliglustat, substrate reduction therapy, switch

## Abstract

Three types of enzyme replacement therapies (ERTs) and two substrate reduction therapies (SRTs) are approved for symptomatic patients with type 1 Gaucher disease (GD1). Eliglustat is the second SRT approved, yet the first to be approved as first-line therapy for any adult patients with compatible CYP2D6 metabolizer genotype. Herein we report safety and efficacy data of the first 29 patients switched from ERT to eliglustat from the Gaucher Unit at Shaare Zedek Medical Center (SZMC) between 07/2017 and 06/2022; the median (range) time on ERT was 13 (0.66–30) years, and the median (range) time on eliglustat was 7 (1–52) months. Most patients switched due to oral preference or sub-optimal response to low-dose ERT. Twelve patients stopped eliglustat after a median (range) of 4 (1–18) months; 11 due to adverse events (AEs) and one due to personal request. There were no drug-related serious AEs and no drug-related cardiac events. Most AEs were mild and transient, mainly dyspepsia. Efficacy achievements were reflected by maintaining stability. We concluded that switching from ERT to eliglustat is safe if choosing the appropriate patients. Reassuring patients to tolerate early AEs may reduce discontinuation. Following the response and compliance to therapy is important to ensure long-term efficacy.

## 1. Introduction

Gaucher disease (GD), one of the two most common lysosomal glycolipid storage disorders, is considered rare worldwide but quite common in Israel due to the ethnic predilection of Ashkenazi Jews, where the estimated birth frequency is about 1:800 [1]. As the specific Gaucher center that was founded in the early ’90s at Shaare Zedek Medical Center Jerusalem (SZMC), a relatively large number of patients have been exposed to Gaucher-specific therapies, including intravenous enzyme replacement therapies (ERTs) and oral substrate reduction therapies (SRTs).

Eliglustat (Cerdelga™, Sanofi/Genzyme) is the second SRT, which was approved in 2014 for patients with type I GD, but in contrast to miglustat (Zavesca™, Johnson & Johnson’s), the first SRT, which received both EMA (2002) and FDA (2003) approvals for patients with mild to moderate GD1 for whom ERT was not suitable or not a therapeutic option, eliglustat was approved as first-line therapy to any GD1 patients who have compatible CYP2D6 metabolizer genotypes [2]. In view of an apparent inferior efficacy and a more problematic safety profile, including cardiac adverse events (AEs) in at least 5% of the patients who participated in the clinical trials of eliglustat [3,4], we among other investigators have challenged the justification of eliglustat label indicating first-line therapy and suggested to use it as a second-line therapeutic option for patients with GD1 who are unwilling or unable to receive ERT [4,5]. This approach has been criticized by a greater number of investigators who consider eliglustat to be equal to ERT [6,7].

The Israeli Ministry of Health approved eliglustat in 2017, using an identical label as the one approved by the FDA; nevertheless, due to the higher cost of this SRT compared to the three commercially available ERTs based on a low/medium dose regiment (30/60 U/kg EOW) (Supplementary Table S1), the majority of the patients approved by the Health Care Scheme to receive eliglustat were those who had medical indications beyond a personal preference to receive oral medication [8]. The purpose of this report is to describe the real-life safety and efficacy data of our first 29 GD1 patients who have been approved for the switch from ERT to eliglustat.

## 2. Methods

All adult patients (age 18 and above) with GD1 followed at the Gaucher Unit in SZMC, who have been switched from any ERT to eliglustat between July 2017 and June 2022, and received at least a single dose, were included. Six patients had a gap of several months from discontinuation of ERT before the actual beginning of eliglustat, mainly due to the time interval required to receive approval for eliglustat and unwillingness to continue with ERT. The time range being between 7 months and 24 months. The safety analysis, covering all patients in this cohort, included all reported AEs defined as severe, moderate, and mild based on physician decision. The rate, timing, and reason for discontinuing eliglustat were recorded. Efficacy measures were reported only for the 12 patients who switched immediately from ERT to eliglustat and for whom data from all three time-points-a. pre: the clinic visit at least six months before the switch, b. baseline: the clinic visit less than six months before the switch, c. post: the clinic visit at least six months after the switch, were available. Efficacy parameters included changes in platelet count, plasma levels of hemoglobin, glucosylsphingosine (lyso-Gb1), HDL cholesterol and ferritin, liver and spleen volumes, and bone density (T score of lumbar spine measures by density dual-energy X-ray absorptiometry (DEX)). All efficacy parameters were analyzed locally, whereas lyso-Gb1 was performed at Centogene (Rostock, Germany) as previously reported [9]. IRB approval was obtained for this retrospective audit.

### Statistical Analysis

To report summary descriptive statistics, we used median and range. Frequencies were used for categorical data. Due to the small sample size, non-parametric tests were used for analysis. The Mann-Whitney U Test was used to compare those with or without efficacy data. The Friedman test was used to compare non-parametric repeated paired measures, i.e., pre, baseline, and post. The Wilcox test was used to calculate pairwise comparisons between group levels with corrections for multiple testing. R programming (RStudio 2022.02.1+46) was used for analysis. *p* value < 0.05 was considered significant.

## 3. Results

Twenty-nine adults with GD1 received at least one dose of eliglustat during the study period (Table 1).

SZMC, Shaare Zedek Medical Center; GBA1, glucocerebrosidase; ERT, enzyme replacement therapy; SRT, substrate reduction therapy. The Mann-Whitney U Test was used to compare those with and without efficacy data.

### 3.1. Safety

The most common AEs were upper gastrointestinal symptoms, including heartburn and dyspepsia (Table 2). All AEs were mild or moderate in severity and transient in nature. Nevertheless, it was the most frequent reason for eliglustat discontinuation; nine patients did not tolerate the AEs and decided to stop their own treatment, and two additional patients with similar AEs, consulted their physician before discontinuation (one due to lack of efficacy and the other due to her desire to become pregnant). Another patient stopped eliglustat, when miglustat became available as a personal choice. The median (range) time on eliglustat before discontinuation was 4 (1–18) months.

### 3.2. Efficacy

Efficacy data was available after a median (range) of 14.5 (6–52), mean (SD) of 21.5 (15.9), months for 12 patients (Table 3). The clinical characteristics of the patients with efficacy data were not different from the entire cohort (Table 1). The median platelet count, hemoglobin, HDL, and ferritin levels, available for all patients, were not significantly different in the post-switch visit compared to baseline and pre-switch visit. Spleen and liver volume and the T-score of the lumbar spine, also did not change significantly over time. The actual changes are presented in Supplementary Table S2.

**Figure 1:**
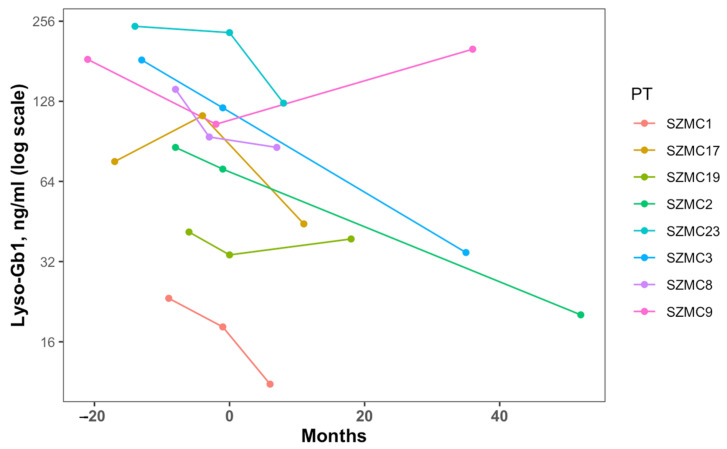
Change over time in lyso-Gb1 levels. Time 0 is the time of the switch.

The lyso-Gb1 levels continued to decrease during the follow-up from a median of 114 ng/mL to 41.6 ng/mL. The difference between pre and baseline levels and between baseline and post-levels were nonsignificant. However, different responses were seen for individual cases (Figure 1).

## 4. Discussion

Switching from intravenous ERT to the oral SRT eliglustat, beyond case reports, has so far been reported almost exclusively by the manufacturers of the drug along with investigators from the various trial sites or the Sanofi-sponsored registry ICGG [10,11]. Hence, this paper of real-life experience of patients with GD1 who switched from any ERT to eliglustat at SZMC in Jerusalem is important even for the mere fact that it has not been written by a medical writer sponsored by Sanofi, nor has it been supported by a grant from the manufacturers; it is representing the group that has expressed concerns about the justification of labeling eliglustat as first-line therapy [4]. In contrast to the real-world report from the ICGG, wherein Sanofi employees designed the study, performed its analysis, and reviewed all data for accuracy, in our paper, study design, data collection, review, and analysis, were conducted by independent researchers. This difference in the methodology of the study explains in part the lack of adverse events (AEs) reports and the lack of information about the causes for drug discontinuation among 10% of the patients in the ICGG paper (an important reflection of drug safety if the reason for stoppage was an AE) [10]. In fact, even this 10% discontinuation rate (in our current report it was 37%) is significantly greater than for any ERT reported so far. For example, Deegan and Cox reported in 2012 that only 3 of 262 (1.1%) patients discontinued imiglucerase due to AE [11]. A study from Japan showed that only one of 53 patients, including all three types of GD, stopped Velaglucerase alfa due to an AE (1.9%) [12]. These numbers support our basic approach towards eliglustat as a second-line therapy as previously discussed [5].

Nevertheless, it should be noted that the higher percentage of discontinuation at the Gaucher Unit in SZMC, compared to the ICGG report reflects the greater safety concern of our group. There are two main take-home messages derived from this report, the first, encouraging the patients to give this SRT a chance before stopping, by using proton pump inhibitors (PPIs), to overcome the heartburn during the early days and weeks of eliglustat may eventually help the patients benefit from this oral option. The importance of careful selection of the most suitable candidate patients by pre-screening with ECG and Holter monitoring to exclude underlying cardiac findings and by consulting our cardiologist expert any time there is a concern in order to avoid life-threatening cardiac AEs. Indeed, in contrast to the previous safety report by the manufacturer [3] we have not seen any cardiac AEs, not even palpitations and syncope.

Other explanations to the better cardiac-related safety profile of our cohort are yet the much smaller number of patients, and the pre-testing of the CYP2D6 genetic status, which has not been part of the inclusion criteria during the drug development program.

There are two additional differences between our cohort and the ICGG report [13], the first is the fact that the majority of our patients were treated with a low-dose ERT regimen (typically 15–30 units/kg body weight (BW) every other week (EOW)), whereas the majority of the patients from the USA received the high-dose 60 units/Kg BW/EOW regimen. One could claim that this dosing difference accounts for the sub-optimal response to ERT as a cause for switching. This interesting consideration has not been evaluated systematically in the literature with the exception of the negative impact of multiple focal splenic lesions particularly on the platelets response [14]. In the current report, among the 9 patients who switched because of sub-optimal response, only 3 were on the low-dose, 4 were on the 30 units/kg EOW, and another two patients were on the high-dose of 60 units/kg EOW, and finally in the ICGG paper [15], wherein most of the patients have been on high-dose ERT, still 1/3 of them had thrombocytopenia, in the ranges similar to our cohort, suggesting that the reasons of sub-optimal response were not dose related. The second aspect related to the methodology used in our study, as we chose to show three-time points, i.e., pre, baseline and post switch, in order to better assess the changes in GD-disease-related parameters after the switch more accurately, and also to exclude patients who did not switch immediately from ERT to SRT.

Although, some patients improved after the switch, and others deteriorated (Supplementary Table S2), our data confirms that in the majority of the patients, switching from ERT to SRT maintained stability in the key disease parameters. Variable responses were also seen by Kleytman et al. [12], however, in this study, the improvements in spleen volume, platelet count, chitotriosidase, and lyso-Gb1 following the switch seems to be more significant. Since the characteristics of the patients in the two studies are rather similar, we speculate that the differences in the results could be explained by the different nature of the report. Our study reflected a real-life scenario, whereas the Yale study was conducted as a clinical trial, i.e., patients were asked to sign consent prior to the switch to eliglustat. Being part of a clinical trial, especially for daily oral therapy, is crucial for better compliance and hence better results. An alternative explanation might be the use of only two time-points, before and after switch, which corresponds to our baseline and post switch data, missing the third time-point “pre” included in our analysis. Pre baseline deterioration can lead to a false impression of improvement.

And additional issue that requires consideration, is the confusion related to the assessment of efficacy by “maintaining stability”. This new definition was introduced for the first time in the ENCORE phase 3 switch-over trial [16]. Whereas all previous switchover trials (from imiglucerase to miglustat [17,18,19], to velaglucerase alfa [20,21] or taliglucerase alfa [15] have used “lack of deterioration” to reflect stability among patients already treated with the commercial ERT [4]. Without reviving an old argument related to this definition [4], which we are keeping in our current report, one should keep in mind that if the baseline value is abnormal, maintaining stability should not be considered a success, and in fact, in the case of very low platelet count, for example, the treating physician should seek an alternative therapeutic modality or another drug.

The main limitations of our report are derived from the nature of real-life data, as they cannot replace accurate time-points and uniform methodologies occurring in multi-center clinical trials, and the relatively small number of patients reported herein, reflects in part the difficulties to obtain approval for the switch from ERT to the more expensive eliglustat in Israel, where there is no specific medical indication for the change (patient preference is not always accepted).

While the existence of different treatment options for GD1 is a blessing, it may also raise confusion to both patients and physicians [5], and at times an inappropriate choice might be harmful. The lesson learned from our real-life data presented here, is that choosing the right patients for the switch would lead to a safe and effective outcome beyond merely the improvement in quality of life gained by the oral medication versus intravenous infusions.

## Figures and Tables

**Table 1:** Clinical characteristics of patients with GD1 switching to eliglustat.

	Total	Efficacy Data	
		Yes	No
Number	29	12	17
Sex, male (%)	18 (62)	9 (60)	11 (65)
Age (years) at the time of switch *	39 (18–70)	44 (18–62)	28 (18–70)
*GBA1* variants ** (%) - N370S/N370S - N370S/Other - Other/Other	13 (45) 12 (41) 4 (14)	7 (58) 5 (34) 2 (13)	6 (35) 7 (50) 2 (14)
Splenectomy (%)	3 (10)	2 (16.5)	1 (6)
Years on ERT *	13 (0.7–30)	13 (3–30)	10 (0.6–29)
Monthly dose of ERT Units/kg (%) - 30 - 60 - 120	17 (59) 9 (31) 3 (10)	7 (58.5) 4 (33.5) 1 (8)	10 (59) 5 (29) 2 (12)
Reason for switching to SRT (%) - Adverse events to ERT - Patient request - Sub-optimal response to ERT	2 (7) 18 (62) 9 (31)	0 (0) 7 (58) 5 (42)	2 (12) 11 (65) 4 (23)
Time on SRT (months) *	7 (1–52)	14.5 (6–52)	3 (1–18)

* Median (range). ** Old nomenclature.

**Table 2:** Adverse events associated with eliglustat.

Adverse Events	Number of Patients * (%)
Gastrointestinal-Heartburn/Dyspepsia	14 (48%)
Chest pain	7 (24%)
Cough/Dyspnea	3 (10%)
Urticaria	4 (14%)
Other allergic reactions (Shortness of breath and dysphagia)	2 (7%)

* A patient may have more than one AE reported.

**Table 3:** Changes in clinical and laboratory parameters between the three-time points- Pre, at least 6 months before the switch; Baseline; less than 6 months before the switch; Post, at least 6 months after the switch.

	Patients	Pre	Baseline	Post
Time from switch, months	12	−9 (−21 to −6)	−1 (−0 to −4)	+14.5 (+6 to +52)
Weight, Kilogram	12	79.5 (47.9−101.4)	74.3 (48.5−101.9)	85 (48−102)
Hemoglobin, gr/dL	12	14.15 (9.9–17)	14.9 (11.1–16.1)	14.1 (10.9–16.3)
Platelet count, ×10^9^/L	12	146 (34–312)	155 (24–396)	149 (34–377)
HDL, mg/dL	12	34 (21–55)	34 (26–54)	35 (25–55)
Ferritin, ng/ml	12	218 (37–1297)	276 (44–1177)	209 (35–1056)
Lyso-Gb1, ng/mL	8	114 (23.3–245)	99.5 (18.2–232)	41.6 (11.1–201)
Spleen volume, × MN	11	5.6 (4.5–19.9)	5.8 (4.3–21.7)	5.4 (4.2–16.9)
Liver volume, × MN	12	1.1 (0.9–1.4)	1.2 (0.9–1.4)	1.2 (1–1.4)
T score, lumbar spine *	10	−1.65 (−3.2 to 0.1)	−1.1 (−3.2 to 1)	−1.55 (−3 to 0.6)

Results are presented as median (range). MN, Multiple of normal. * Not all patients had the three-time points. The Friedman test was used to compare the pre, baseline, and post paired measures.

## Data Availability

Data cannot be shared due to ethical and privacy issues.

## Supplementary Materials

The following supporting information can be downloaded at: https://www.mdpi.com/article/10.3390/jcm11216265/s1, Table S1: Cost of Gaucher disease- specific therapy according to Israeli Ministry of Health (MoH) reports; Table S2: Gaucher disease-related parameters pre, baseline and post switch.
