# Supplementary material for: Real-Life Experience with Oral Eliglustat in Patients with Gaucher Disease Previously Treated with Enzyme Replacement Therapy
Source: J Clin Med. 2022 Oct 24;11(21):6265. doi: 10.3390/jcm11216265 (PMC9659144; doi:10.3390/jcm11216265)
Supplement: Supplementary file 1 [file jcm-11-06265-s001.zip › jcm-1908533-supplementary.pdf]

Journal of
Clinical Medicine

MDPI

Table S1. Cost of Gaucher disease- specific therapy according to Israeli Ministry of Health (MoH) reports. Prices are listed in NIS, and based on the MoH prices reports from 30.12.2021 (https://www.health.gov.il/Subjects/Finance/DrugPrice/Pages/default.aspx).

Drug	Monthly Cost for a 70 kg Patient Receiving a Low Dose of ERT (30 U Monthly)	Monthly Cost for a 70 kg Patient Receiving a Medium Dose of ERT (60 U Monthly)	Monthly Cost for any Adult Patient Receiving 2 Capsules a Day
Imiglucerase	29,816.9	65,597.18	
Velaglucerase alfa	32,551.85	71,614.07	
Taliglucerase alfa	Not reported	Not reported	
Eliglustat			87,443.35
Miglustat			19,458.42

Table S2. Gaucher disease-related parameters pre, baseline and post switch.

No./	Hemoglobin, g/dL			Platelet Count, ×10³/µL			HDL Cholesterol, mg/dL			Ferritin, ng/mL		
Sex	Pre	Baseline	Post	Pre	Baseline	Post	Pre	Baseline	Post	Pre	Baseline	Post
1/M	14.3	14.9	14.6	94	100	100	36	33	36	197	183	191
2/M	15.1	15	15.6	109	100	131	36	34	35	313	344	155
3*/M	15.1	13.8	14	302	319	377	33		35	260	335	322
5/F	9.9	11.1	10.9	34	24	34	21	34	34	849	657	686
8*/F	13.5	13.4	12.9	312	272	248	29	28	29	209	208	227
9/M	14.9	15	14.4	157	171	128	33.8	36	37	288	276	307
12/F	13	13.1	12.9	305	369	355	51	54	55	150	121	35
14/M	14	15.3	15	183	184	181	36	47	44	37	44	50
17/M	17	16.1	16.3	180	155	170	34	38	46	183	171	149
19/F	11.7	11.5	11.5	112	104	118	26	26	25	228	335	420
20/F	13.4		12.5	119		103	48		44	37		53
23/M	14.5	14.9	14.2	135	121	168	29	31	33	1297	1177	1056

* Splenectomized. M, Male; F, Female; Hb, Hemoglobin; Plt, Platelet count.
